# Discovery of a novel potent peptide agonist to adiponectin receptor 1

**DOI:** 10.1371/journal.pone.0199256

**Published:** 2018-06-18

**Authors:** Sunghwan Kim, Younho Lee, Jun Woo Kim, Young-Jin Son, Min Jung Ma, Jee-Hyun Um, Nam Doo Kim, Sang Hyun Min, Dong Il Kim, Brian B. Kim

**Affiliations:** 1 New Drug Development Center, Daegu-Gyeongbuk Medical Innovation Foundation, Daegu, South Korea; 2 R&D center, Polus Inc., 32 Songdogwahak-ro, Yeonsu-gu, Incheon, South Korea; 3 College of Pharmacy and Yonsei Institute of Pharmaceutical Sciences, Yonsei University, 85 Songdogwahak-ro, Yeonsu-gu, Incheon, South Korea; 4 Department of New Drug Discovery, Samhyun Inc., Daegu, South Korea; 5 Department of Biochemistry, College of Medicine, Dong-A University, Busan, South Korea; 6 Department of Biological Engineering, Inha University, Incheon, South Korea; 7 R&D center, EncuraGen Inc, Anyang, Gyeonggi-do, South Korea; Sungkyunkwan University, REPUBLIC OF KOREA

## Abstract

Activation of adiponectin receptors (AdipoRs) by its natural ligand, adiponectin has been known to be involved in modulating critical metabolic processes such as glucose metabolism and fatty acid oxidation as demonstrated by a number of *in vitro* and *in vivo* studies over last two decades. These findings suggest that AdipoRs’ agonists could be developed into a potential therapeutic agent for metabolic diseases, such as diabetes mellitus, especially for type II diabetes, a long-term metabolic disorder characterized by high blood sugar, insulin resistance, and relative lack of insulin. Because of limitations in production of biologically active adiponectin, adiponectin-mimetic AdipoRs’ agonists have been suggested as alternative ways to expand the opportunity to develop anti-diabetic agents. Based on crystal structure of AdipoR1, we designed AdipoR1’s peptide agonists using protein-peptide docking simulation and screened their receptor binding abilities and biological functions *via* surface plasmon resonance (SPR) and biological analysis. Three candidate peptides, BHD1028, BHD43, and BHD44 were selected and confirmed to activate AdipoR1-mediated signal pathways. In order to enhance the stability and solubility of peptide agonists, candidate peptides were PEGylated. PEGylated BHD1028 exhibited its biological activity at nano-molar concentration and could be a potential therapeutic agent for the treatment of diabetes. Also, SPR and virtual screening techniques utilized in this study may potentially be applied to other peptide-drug screening processes against membrane receptor proteins.

## Introduction

Diabetes mellitus is a type of metabolic disorders exhibiting high blood sugar level, caused by failure to produce enough insulin (type I diabetes) or failure to respond to insulin properly (type II diabetes). Among more than 400 million people with diabetes, about 90% is type II diabetes [1]. Adiponectin, an adipokine produced by adipocytes, has been extensively studied for its antidiabetic, anti-inflammatory, anti-obesity, and cardio protective effects over the last two decades. It exerts its biological functions *via* binding to AdipoR1 and/or AdipoR2 in various cell types including hepatocytes, endothelial cells, pancreatic β cells, and cardiac myocytes [2, 3] and subsequently by activating various signal transduction pathways such as AMP kinase (AMPK), acetyl coenzyme A carboxylase (ACC) and peroxisome proliferator-activated receptor γ (PPARγ) pathways. AdipoR1 is a major adiponectin receptor, which is abundant in skeletal muscle, liver, heart, and kidney tissues [4]. Upon activation of AdipoR1, various signal transduction pathways are activated and changes in the physiological activities of cells such as alleviation of insulin-resistance and suppression of gluconeogenesis and lipoic gene expression are induced. This kind of activation may increase the uptake of glucose as well as the oxidation of fatty acids [5].

The plasma concentration of adiponectin in healthy individuals is 5–30 μg/mL, approximately 0.01% of the total plasma proteins. In the case of obesity, the level of blood adiponectin is generally lower than healthy individuals, and type II diabetes is eventually developed as insulin resistance increases. Supplement of adiponectin ameliorated insulin resistance and glucose intolerance in mice [6–8]. Adiponectin-overexpressing mouse also proved that adiponectin can protect diabetic phenotype or metabolic challenges [5, 9–11]. Based on these findings adiponectin has been suggested as a potential target for developing a therapeutic agent for the treatment of diabetes, especially type II diabetes and other obesity-associated diseases [3]. However, preparation of recombinant adiponectin has been challenged due to post-translational modifications and oligomerization [12, 13] and thus far, no drug targeting AdipoRs has been approved.

Recently an adiponectin-mimetic peptide, ADP355 and a small molecule, AdipoRon, have been developed as agonists to AdipoRs [14, 15]. ADP355 is the first synthetic adiponectin-mimetic peptide containing non-natural amino acids [15, 16]. Although ADP355 has been under development for cancer treatment, its ability to activate AdipoRs-mediated AMPK pathway provided the opportunity that synthetic peptide could provide a possibility to develop agonist against AdipoRs. Moreover, recent studies elucidated high resolution structure of AdipoR1, which opened door to further understand the mechanism of AdipoRs and also allowed structure-based drug design [17–19]. Full length and N-terminally truncated variants of AdipoR1 were successfully expressed in insect cell and purified for crystallographic structure study [18]. Among variants, AdipR1-Δ88, whose structure has been solved, was not aggregated like full-length AdipoR1, so it was identified as an ideal construct to carry out biochemical or biophysical evaluations.

Although the adiponectin and AdipoR1 bound-structure is not available, it is possible to predict their binding interface through computational modeling along with the proposed the minimal active site of adiponectin K149 to T165 segment (KFHCNIPGLYYFAYHIT) and biochemical experiments [15]. In the case of AdipoR1, the extracellular C-terminal domain has been identified to be critical for adiponectin binding [10]. However, recent crystal structure of AdipoR1 suggest to broaden interface at the extracellular regions in addition to the flexible C-terminal tail [17]. These findings led us to identify the ligand-binding regions in AdipoR1 and to design each peptides as well as to simulate interaction between peptide and AdipoR1. Designed peptides were preliminarily screened by assessing their binding ability to recombinant AdipoR1-Δ88 using surface plasmon resonance, and the selected peptides were evaluated for phosphorylation of AMPK in differentiated C2C12 myotubes. Through several rounds of designing and screening, three 15-mer peptides were selected. The selected peptides BHD42, 43, and 1028 have been PEGylated in order to increase their stability and solubility. Among them, PEGylated BHD1028 was identified the most potent peptide in activating AMPK the most, providing the opportunity to become a strong drug candidate. This study also provides an attractive time-efficient strategy to develop synthetic agonists for diseases-related membrane receptors, such as G-coupled protein receptors.

## Materials and methods

### Computational docking simulation

The adiponectin binding to AdipoR1 was analyzed with its recently reported crystal structure (PDB ID: 3WXV) and the configuration of the binding site was applied to the peptide binding simulation study. The possible interaction configurations between AdipoR1 and the designed peptides were analyzed by using Maestro 10.1 and ZDOCK protein-protein docking program in Discovery Studio 4.1. Protein rigid-body docking was performed with extracellular region of AdipoR1 and peptides based on ZDOCK algorithm. The clustering poses were obtained according to the ligand positions, followed by selection of the peptide binding pose that occupied the predicted binding cavities in the extracellular region of AdipoR1. The obtained AdipoR1 and peptide binding model was further optimized by Protein Preparation Wizard in Meastro 10.1 performing a restrained minimization that allow hydrogen atoms to be freely minimized. The minimized protein-protein structures were analyzed to find important interactions. After collecting information about non-bonding interaction, electrostatic interaction and hydrogen bond network between AdipoR1 and peptides, twenty five (25) peptides were selected and further screened by biophysical and biological assays.

### Peptides and recombinant protein

Designed peptides and ADP355 (H-DAsn-Ile-Pro-Nva-Leu-Tyr-DSer-Phe-Ala-DSer-NH_2_) has been chemically synthesized by AnyGen Ltd. (South Korea). Lyophilized peptides were dissolved with distilled water to be more than 2 mM stock concentration, followed by dilution to working concentrations with an appropriate buffer. N-terminal site-specific mono-PEGylation of the selected peptides was accomplished by using PEG-5000 derivative through a reactive terminal aldehyde group as described by Na et. al. [20]. PEGylated peptides were further purified by using a C18 reverse-phase HPLC column. Recombinant human globular adiponectin was purchased from R&D Systems (MN, USA).

DNA gene fragment encoding AdipoR1 fused to an N-terminal flag tag was synthesized after codon optimization for insect cell expression (Integrated DNA Technologies). Full length AdipoR1 and AdipoR1-Δ88 were expressed in SF9 cell line using Bac-to-Bac Baculovirus system and purified following the procedure described by T. Tanabe *et*. *al*. [18]. Briefly, 1 L SF9 cells infected with amplified baculovirus were cultured in SF-900 III SFM media (Thermo Fisher Scientific) for 3 days at 27°C. Harvested cells were disrupted by dounce homogenization, followed by centrifugation at 30,000 rpm for 2 hours. Collected raw membrane were washed with HEPES buffer and stored in -80°C after flash-freezing. Membranes were solubilized with 1% DDM (Anatrace) and recombinant AdipoR1-Δ88 was purified by using anti-flag tag antibody magnetic beads (Sigma). Eluted AdipoR1-Δ88 by flag peptide (Sigma) was further purified in 20 mM HEPES (pH7.4), 150 mM NaCl, 10% glycerol, 0.025% DDM, and 0.0001% CHS by Superdex 200 (GE Healthcare). The amount of purified AdipoR1-Δ88 was estimated by using Bradford staining.

### In vitro binding assay using surface plasmon resonance

In order to screen peptide library against AdipoR1, purified AdipoR1-Δ88 was immobilized onto S-series CM5 chip (GE Healthcare Life Science). Since AdipoR1-Δ88 was solubilized with a detergent-containing buffer solution, pH scouting solutions with various pH from 3.5 to 5.5 were prepared with the same detergent solution, 0.025% DDM and 0.0001% cholesteryl-hemi-succinate (CHS, Anatrace). At pH 5, AdipoR1-Δ88 was immobilized at around 4000 response unit using amine coupling method though EDC/NHS. After enough stabilization with running buffer consisting of 20 mM HEPES (pH7.4), 150 mM NaCl, 10% glycerol, 0.025% DDM, and 0.0001% CHS, serial concentrations from 1 to 100 μM were injected into reference and sample cells with 60-second contact time, 200-second dissociation time, and 30 μl/min flow rate at 4°C. The resulting sensorgrams were double-blanked by reference cell and buffer injection.

### Cell culture and differentiation

Mouse C2C12 myoblasts (American Type Culture Collection) were maintained in Dulbecco’s modified Eagle’s medium (DMEM) supplemented with 10% FBS. C2C12 myotube was obtained by culturing myoblasts in DMEM containing 2% horse serum for at least 5 days with daily media change. On the fifth day, differentiation of cells could be confirmed by multinucleated contracted myotubes. C2C12 myotubes were then treated with selected peptide candidates at indicated concentrations for 30 minutes. As a positive control, 2 μg/ml recombinant human globular adiponectin and indicated concentrations of ADP355 were used.

### Western blot and measurement of AMPK activities

Cell lysates were subjected to SDS-PAGE and western blot analysis. Briefly, cells were lysed with protein lysis buffer (RIPA, Thermo) followed by heat denaturation. Five micrograms of whole cell proteins were applied to SDS-PAGE. After electrophoresis, the proteins were transferred onto PVDF membrane, followed by membrane blocking in TBS-T buffer containing 5% non-fat dry milk for 1 hour at room temperature. The membrane was probed with the following different primary antibodies: anti-phosphorylated-AMPK (Thr172) (Cell Signaling), anti-tAMPK (Cell Signaling), anti-pACC, and anti-GAPDH (Santa-Cruz). Then, the membrane was washed and incubated with peroxidase-conjugated secondary antibody and finally visualized using Chemiluminescent HRP Substrate reagent (Millipore MA, USA) in Gel-Doc imaging system (Bio-Rad). Signals from Western blot were quantified by using Image Lab Software 6.0 (Bio-Rad).

## Results and discussions

### Prediction of ligand-binding regions

**Fig 1:**
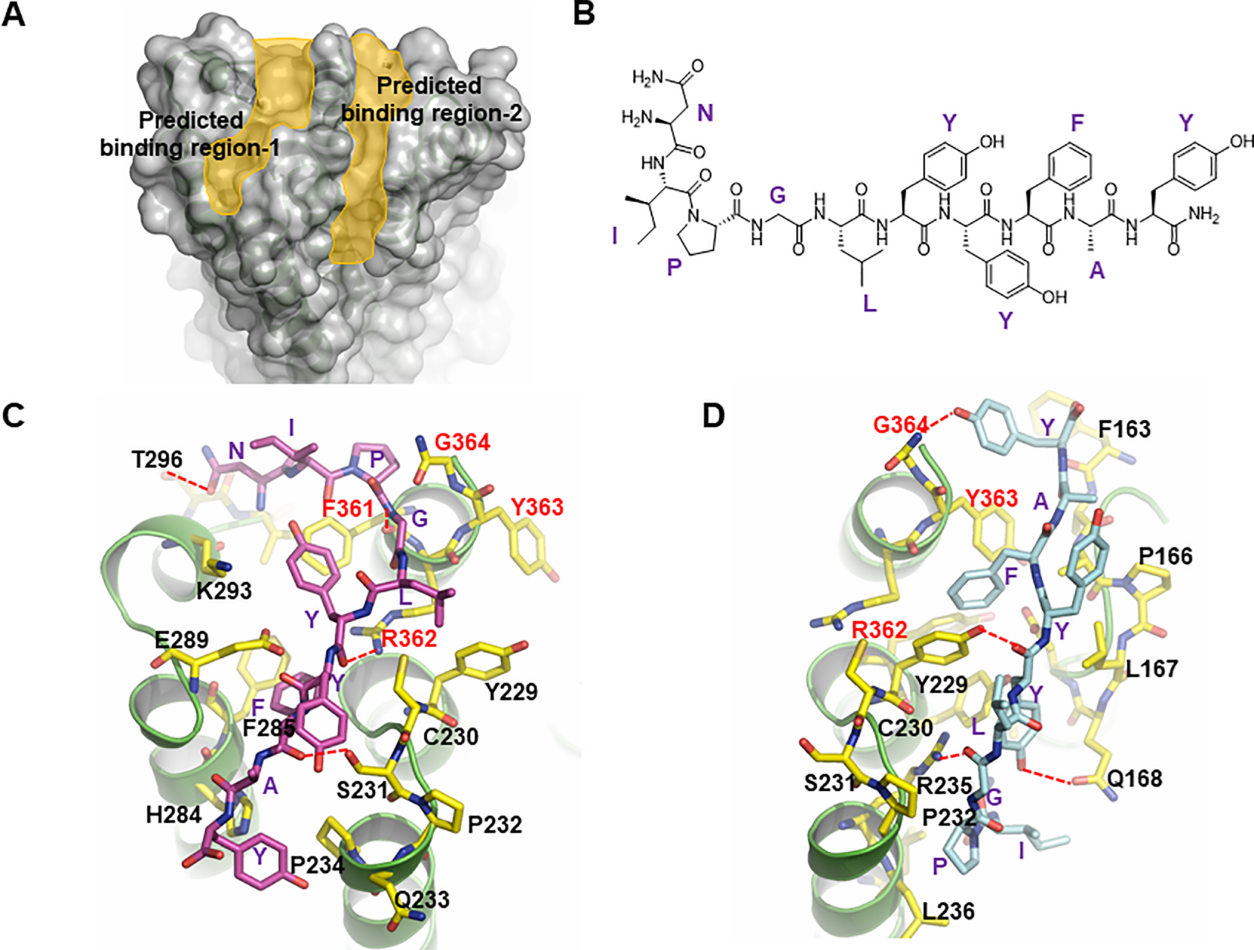
Structural analysis for ligand-binding regions of AdipoR1. A. Adiponectin docking model. Two predicted ligand-binding regions were indicated by yellow colour. For docking analysis, PDB ID 3WXV was used. B. Sequence and structure of active region of adiponectin. C and D. Z-Dock (protein-protein docking) and refinement. Two binding regions (C, site 1 and D, site 2) were separately presented with adiponectin active peptide sequence.

Human adiponectin receptor is a seven-transmembrane protein with N-terminal cytoplasmic domain, so AdipoR1 has three extracellular loops (ECL) and extracellular C-terminal region. Biochemical evaluation results indicated that the C-terminal extracellular region is critical for ligand binding, but recent structural and mutational studies suggest that the conserved extracellular loop residues are also involved in adiponectin signaling [10, 17]. In order to design a structure-based ligand, the docking site of the receptor must be precisely examined. We simulated the interaction between AdipoR1 (Fig 1A) and adiponectin-derived active peptide sequence (NIPGLYYFAY, Fig 1B) through docking modeling and discovered two narrow cavities in the extracellular regions, where two molecules of the active peptide were expected to separately bind in opposite directions. The C-terminal region (F361 to G364) and ECL2 (Y229 to R235) (Fig 1C and 1D) centered two binding sites, of which the first binding site (Fig 1C) and the second binding site (Fig 1D) face ECL3 and ECL1, respectively. The results of simulation study supported extended ligand binding interface formed by all extracellular loops and C-terminal region as suggested by previous study [17]. Considering oligomerization of adiponectin [21], it would be possible that two adiponectin molecules bind to one AdipoR1. The discovery of two opposite-direction binding sites provided invaluable information in designing a peptide library.

### Structure based peptide design and screening

We initially referenced predicted active sequences of natural ligands such as human adiponectin (NIPGLYYFAYHP, Fig 1B) and Osmotin (CTQGPCGPT), which have also been known to activate AdipoR1 [22]. Along with the active sequence information of the natural ligands, the local structure of predicted ligand-binding regions were examined. They were subsequently evaluated from the perspective of binding capability of each peptide docks to AdipoR1 in a structurally and chemically complementary manner. Then the peptides were further assessed for physical interaction of the peptides to AdipoR1, and finally the selected peptides were evaluated by assessing the level of AdipoR1 phosphorylation on cells.

**Fig 2:**
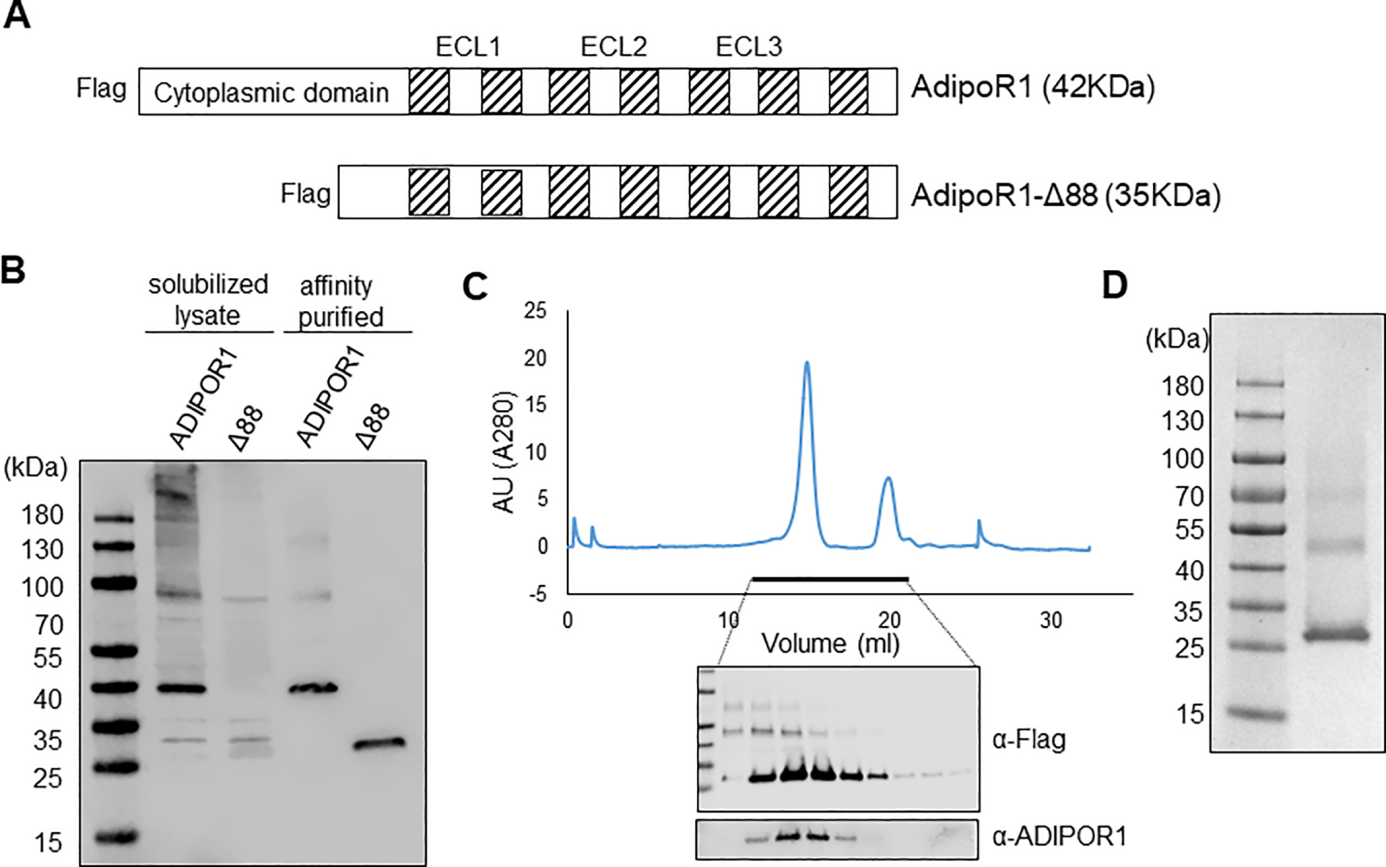
Preparation of active AdipoR1. A. Structural diagram for Adiponectin receptor 1 and its deletion mutant, Δ88. Shaded regions indicate transmembrane domain. ECL1, ECL2, ECL3, and c-terminal domain form extracellular surface. N-terminal 88 residues were deleted in AdipoR1-Δ88. Both constructs have N-terminal flag tag for further purification. B. Over-expressed recombinant AdipoR1 and AdipoR1-Δ88 in insect cells were analyzed before and after anti-flag affinity purification by Western blot with anti-flag antibody. C. AdipoR1-Δ88 purified by anti-flag beads were further purified by size exclusion chromatography to remove other impurities and flag peptide and to replace buffer. Eluents were analyzed by Western blot with anti-flag and anti-AdipoR1 antibodies. D. Purified AdipoR1-Δ88 was analysed by non-reducing SDS-PAGE.

In assessing the physical interaction between AdipoR1 and the designed peptides, we applied surface plasmon resonance (SPR) method that provides kinetic information such as association and dissociation rates besides binding constants. For SPR, human recombinant AdipoR1 and AdipoR1-Δ88 were expressed in insect cells (Fig 2A and 2B), but only purified AdipoR1-Δ88 was used for assay system (Fig 2) because of its benefit as a monomeric form [18] for SPR analysis. Oligomeric forms of AdipoR1-Δ88 were not detected during the preparation (Fig 2C), but oligomeric forms appeared in concentrated sample (Fig 2D). SDS-PAGE analysis showed that adequate preparation of the monomeric AdipoR1-Δ88 of around 25 kDa was adequately prepared (Fig 2D).

While designing peptides, we observed two important factors. First, through docking simulation between ADP355 and AdipoR1, it was found that hydrophobic interaction near the C-terminal domain, especially F361 and Y363, is crucial (Fig 1C and 1D) in both ligand-binding regions. In order to confirm this finding, two analogues of ADP355, Tyr6Ala and Phe8Ala, were synthesized and evaluated for their abilities to activate AdipoR1. As expected, two ADP355 mutants showed lower activity to phosphorylate AdipoR1 than ADP355 in HepG2 cells (S2 Fig). Based on this information, this hydrophobic interaction was maintained throughout designing processes in further peptide design by fixing the hydrophobic tri-peptide motif ɸYF (ɸ: hydrophobic residues).

The second important factor we observed that repeating-ɸYF motifs could increase binding ability and cellular efficacy by binding two ligand-binding regions simultaneously. Although two ɸYF motifs could be a feasible option to achieve higher affinity, we speculated its ability to maintain the stability of the molecule. As a result, the focus of our design concept was narrowed down to a short but effective peptide.

### SPR-based ligand screening system

**Fig 3:**
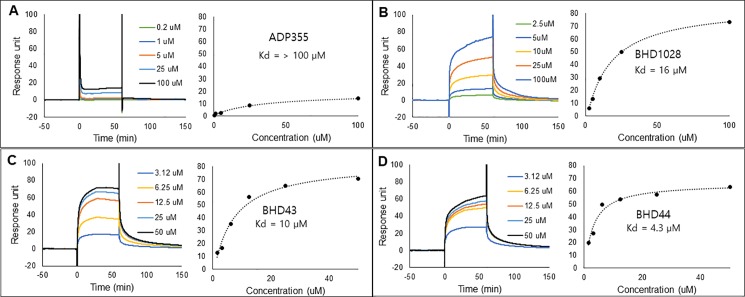
Surface plasmon resonance analysis. ADP355 (A), BHD1028 (B), BHD43 (C), and BHD44 (D) were injected into AdipoR1-Δ88 -immobilized CM5 chip. SPR signals from different concentrations were plotted against concentrations to calculate Kd value.

During the course of the study, we were also able to evaluate the SPR-based testing system for its potential standardized application for peptide screening. This biophysical screening method using SPR allowed to evaluate binding affinity and binding quality of the candidates to AdipoR1 at the same time. A concentration-dependent experiment could give information on the extent of an efficient comparison of the binding affinities of the peptides to the target and minimize false positive hits. An SPR sensorgram itself provided much information to decide hit peptides. Slower dissociation pattern may implicate better efficacy. In fact, among screened peptides, BHD1028 showed the slowest dissociation in the sensorgram and the highest biological efficacy. Purified AdipoR1-Δ88 was successfully immobilized on CM5 by the amine coupling method. In order to maintain the active conformation of the transmembrane protein, DDM and CHS were introduced in immobilization and running buffers. While several previous studies reported cellular efficacy of ADP355 in nano molar concentration [15, 16, 23, 24], ADP355 revealed very weak SPR responses except 100 μM concentration (S1 Fig) in our study. This discrepancy might imply inappropriate *in vitro* system due to artificial environment, such as immobilization of membrane protein on the chip, which differs from cellular membrane. However, some designed peptides showed higher responses in a concentration-dependent response pattern. Moreover, some designed peptides showed slower dissociation rate than ADP355. For example, during early screening stage, BHD15 showed not only stronger SPR response (S1 Fig) but also better capability of phosphorylating AdipoR1 than ADP355 (S2 Fig). This indicates that SPR system with solubilized recombinant AdipoR1-Δ88 could be an efficient way to screen peptides. Because of this reason, ADP355 was included as a positive control for every SPR screening process. Among the designed peptides, three peptides BHD31 (BHD1028), BHD43, and BHD44 were selected based on strength of SPR response, concentration-dependency, and dissociation rate (S2 Fig). These selected peptides were further characterized with SPR to calculate their Kd values (Fig 3 and Table 1). The Kd values of the selected peptides were calculated from saturation curves with responses at equilibrium state. Among the three peptides, BHD44 showed the lowest Kd value of 4.3 μM. Since responses of ADP355 at equilibrium state did not reach near Rmax value, the Kd value of ADP355 to AdipoR1 could not be calculated but estimated to be above 100 μM under the applied SPR system to this study (Fig 3A).

**Table 1:** Comparison of selected peptides to adiponectin and ADP355.

Compounds	Amino Acid Sequence	Kd (μM)
Adiponectin	-Asn-Ile-Pro-Gly-Leu-Tyr-Tyr-Phe-Ala-Tyr-	N.D.
BHD1028	NH_2_-Tyr-Tyr-Phe-Ala-Tyr-His-Pro-Asn- Ile-Pro-Gly-Leu-Tyr-Tyr-Phe-COOH	16
BHD43	NH_2_-Trp-Tyr-Phe-Ala-Tyr-His-Pro-Asn- Ile-Pro-Gly-Leu-Trp-Tyr-Phe-COOH	10
BHD44	NH_2_-Phe-Tyr-Phe-Ala-Tyr-His-Pro-Asn- Ile-Pro-Gly-Leu-Phe-Tyr-Phe-COOH	4.3
ADP355	H-DAsn-Ile-Pro-Nva-Leu-Tyr-Dser-Phe-Ala-DSer-NH_2_	> 100

ADP355: A reference peptide consisting of non-natural amino acids

Adiponectin: Sequence of active region was presented

N.D.: Not Determined

### Docking simulation of hit peptides

**Fig 4:**
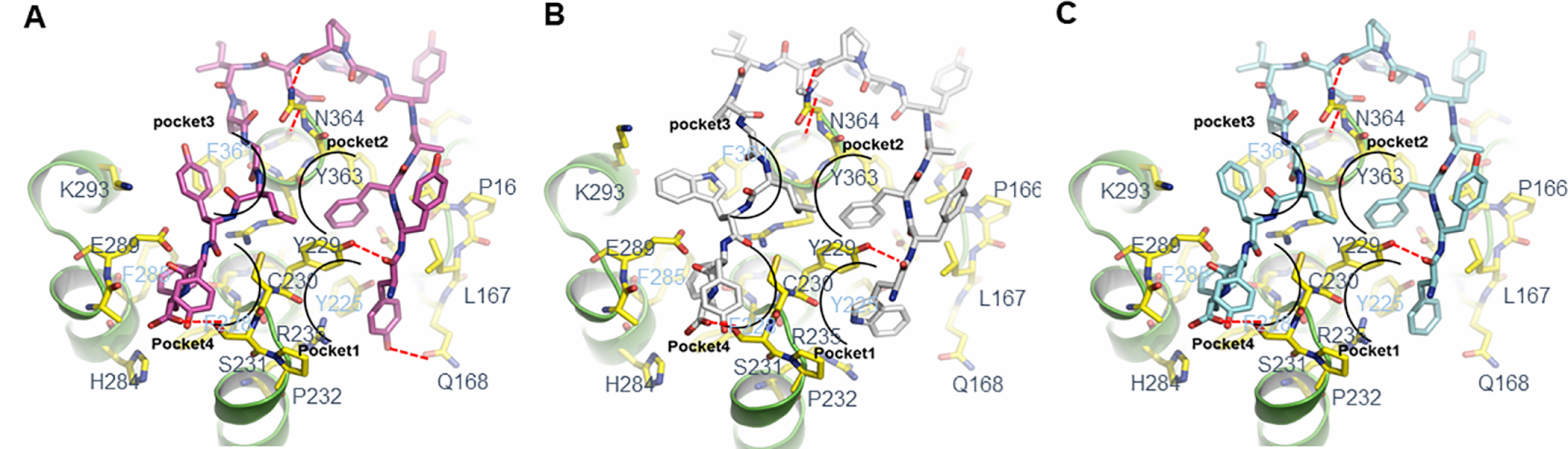
Docking simulation of selected peptides into AdipoR1. BHD1028 (A), BHD43 (B), and BHD44 (C) were simulated to bind to AdipoR1. Four major binding pockets were indicated with arched lines. Pocket 1 and 2 formed ligand binding site 2 and pocket 3 and 4 formed ligand binding site 1. Expected hydrogen bonds were indicated by red dot lines and key residues were annotated with number.

Docking simulation of the selected peptides onto AdipoR1 was followed in order to evaluate their interaction. Based on this simulation, we discovered that each ligand-binding region was further divided into two pockets, resulting in four major binding pockets (Fig 4). All pockets were mainly composed of hydrophobic residues: pocket 1 was composed of L167, Y225, and Y229; pocket 2 was composed of Y229 and Y363; pocket 3 was composed of K293 and F361; pocket 4 was composed of F228 and F285 (Fig 4). BHD1028 is expected to bind to all four major pockets (Fig 4A). The N-terminal Tyr and the third Phe of BHD1028 could form hydrophobic interaction with the pocket 1 and 2, respectively. The 13^th^ Tyr and the 15^th^ Phe could also form hydrophobic interaction with the pocket 3 and 4, respectively. Beside major hydrophobic interactions, the N-terminal Tyr of BHD1028 may form hydrogen bonds with Q168 and Y229. Also, the 7^th^ Pro and the C-terminal carbonyl group of BHD1028 may form hydrogen bond with the side chain of N364 and S231, respectively. BHD43 and BHD44 were also expected to bind to four major pockets like BHD1028 (Fig 4).

Considering structural and functional similarity between AdipoR1 and AdipoR2, the selected peptides against AdopoR1 might also bind to AdopoR2. In order to investigate binding specificity of the selected peptides, docking simulation of BHD1028 against AdipoR1 and AdipoR2 were conducted. As a result, it was found that AdipoR2 has Met375 at the position of Gly364 of AdipoR1, and Met375 may have steric hindrance to the hinge region of BHD1028 peptide (S3 Fig). Therefore, BHD1028 may bind to AdopoR1 more tightly than AdipoR2.

### Activation of adiponectin signalling in differentiated C2C12

**Fig 5:**
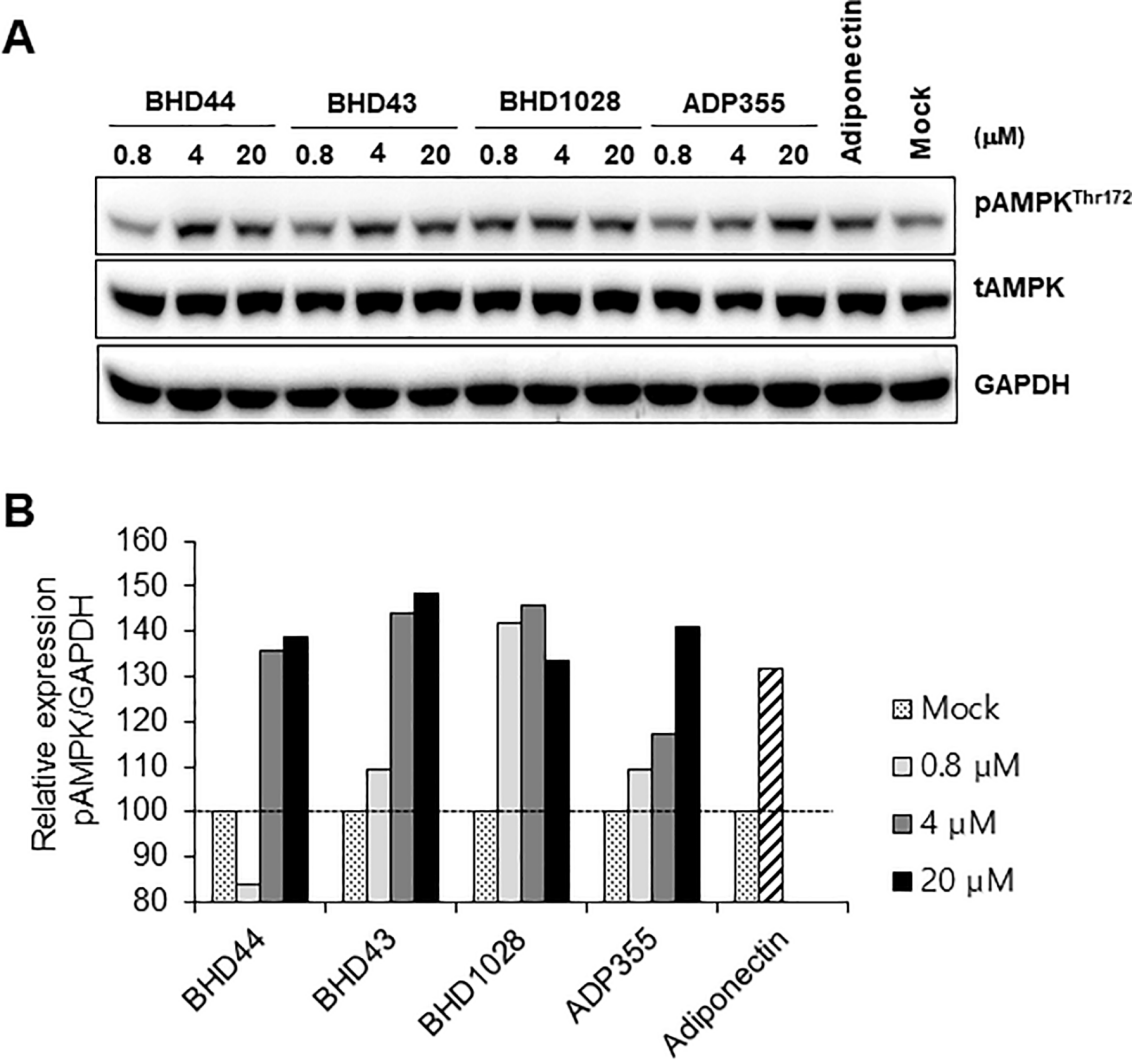
AMPK activation by selected peptides. A. Differentiated C2C12 myotubes were treated with 0.8, 4, and 20 μM of BHD1028, BHD43, and BHD44. As a positive control, ADP355 and 2.5 μg/ml globular adiponectin were treated. AMPK activation was analyzed by Western blot with anti-pAMPK^thr172^, anti-total AMPK, and GAPDH. B. Western blot signals were quantified by densitometer and relative signal were calculated by dividing pAMPK by GAPDH signals.

In order to evaluate the biological activity of selected three peptides, BHD1028, BHD43, and BHD44, AdipoR1-mediated AMPK activation was assessed *via* cell-based assays with differentiated C2C12 myotubes [5]. In the assay, we confirmed that 2.5 μg/ml globular adiponectin and 20 μM ADP355 induced AMPK activation. BHD43 and BHD44 also activated AMPK in a concentration-dependent manner like ADP355 (Fig 5). In the case of BHD1028, AMPK was activated even at 0.8 μM, indicating that BHD1028 may exhibit higher biological efficacy than other peptides including ADP355.

**Fig 6:**
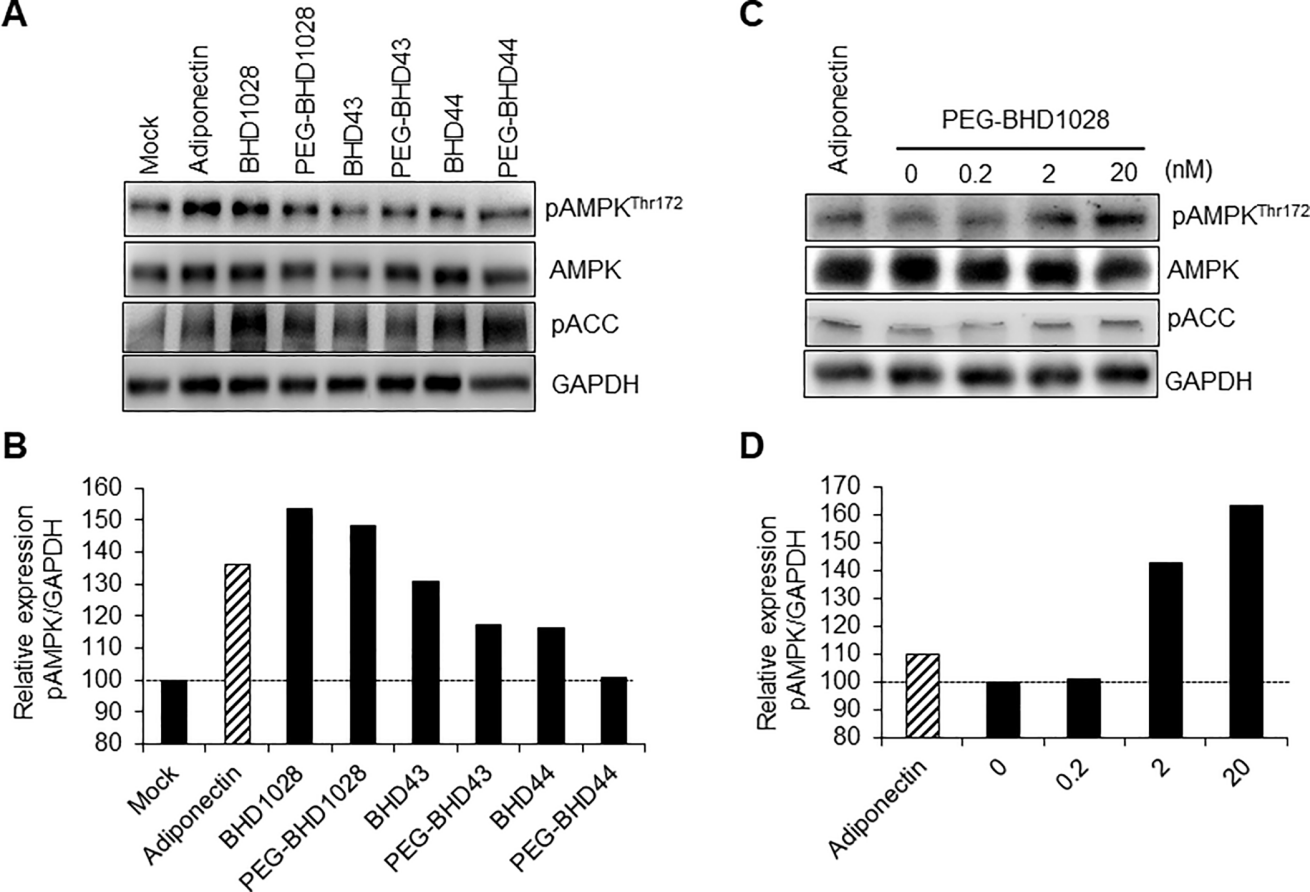
AMPK activation by PEGylated peptides. A and B. Selected peptides and PEGylated peptides treated to differentiated C2C12 myotubes at 20 μM. C and D. PEGylated BHD1028 induced AMPK and ACC activation with concentration dependent manners. Activation of AMPK and ACC were assessed by Western blot analysis (A and C) and their signals were quantified by densitometer (B and D).

In general, the use of peptides as a pharmaceutical drug may be hampered by their extremely short half-life due to proteolysis, and renal and kidney clearance [25, 26]. Also, the selected peptides exhibited low solubility in a neutral physiological pH buffer since they contain a number of hydrophobic residues. For example, BHD1028 was soluble in DMSO up to a concentration of 100 mM, but it was not soluble in PBS buffer (pH 7.4) at concentration more than 50 μM. In order to overcome these concerning aspects of peptides, we modified the selected peptides with 5000 Da polyethylene glycol (PEG) and confirmed that their solubility was enhanced. All three peptides were soluble in PBS buffer at concentration more than 1 mM. Procedures and details for PEGylation of BHD1028 and others will be published elsewhere. Then, we analyzed the impact of PEGylation in biological functions in comparison with the original peptides (Fig 6). At a concentration of 20 μM, PEGylated peptides showed slightly decreased efficacy in activating AMPK. Among the selected peptides, BHD1028 showed the least difference before and after PEGylation (Fig 6). Beside AMPK activation, acetyl Co-A carboxylase (ACC), another signaling marker in adiponectin signal pathway, was also confirmed to be phosphorylated by the selected peptides and their PEGylated forms. Finally, we confirmed that PEGylated BHD1028 could induce AMPK activation in a concentration-dependent manner from 0.2 to 20 nM (Fig 6). These findings suggest that BHD1028 could be a promising drug candidate for the treatment of diseases attributed by a lack of adiponectin in both quantitative and qualitative aspects.

### Peptide drug development for targeting AdipoR1

Adiponectin and adiponectin receptors have been considered as a potential drug candidate and target for metabolic disorders such as diabetes mellitus based on *in vitro and in vivo* studies [5–11]. Several groups have tried to develop drugs to activate adiponectin receptors with recombinant adiponectin, adiponectin-mimetic peptides, or synthetic small molecules [2, 12–15]. However, none of them has been approved yet. In this study, we discovered potent adiponectin-mimetic peptide agonists by using structure-based drug design and time-efficient screening system combining virtual docking simulation, physical interaction screening, and cell-based assay. Virtual docking simulation could generate candidates in a time-efficient manner, and physical-interaction screening could quickly eliminate non-binders or low-affinity binders to provide information for the next round of structure-based drug design without the usage of time-consuming cell-based assay.

In order to develop peptide-based pharmaceutics, it is necessary to overcome low stability and solubility issues. Non-natural amino acids, terminal capping, or amino acid modification could be applied to minimize degradation. In the case of peptides with low solubility like BHD1028, amino acid composition changes or chemical modification such as PEGylation could be applied to increase solubility. Here, we modified candidate peptides with 5000 Da of PEG. As a result, PEGylated BHD1028, BHD43, and BHD44 showed much enhanced solubility. Since PEG-5000 is quite large compared to peptides (1600 Da), PEGylation may reduce the efficacy of peptide by providing steric hindrance. However, we confirmed that PEGylation did not reduce biological efficacy of BHD1028, which was considered as a primary candidate. Although binding affinity of BHD1028 calculated by SPR experiment was 16 μM, AMPK activation was observed even at 20 nM PEGylated BHD1028 in cell-based assay. This big difference could be due to generic difference between biophysical assay and cell-based assay. SPR method used purified AdipoR1 isolated from the membrane to minimize non-specific binding, whereas cells provided complicated environment such as homo-dimerization or complex with other cellular component such as membrane on the cell surface. Otherwise, enhanced solubility and stability by PEGylation might simply increase biological efficacy of peptides. These results indicated that PEGylated BHD1028 could be a potential drug candidate for an anti-diabetes or anti-metabolic diseases.

## Supporting information

S1 Fig Receptor phosphorylation by selected peptides. HepG2 cells were treated with 2 μg/ml globular adiponectin, 10 μM ADP355, ADP355-Y6A, ADP355-F8A and other designed peptide during early screening stage. Activation of AMPK was analyzed by Western blot with anti-pAMPK. Western signal of phospho-AMPK was normalized by that of vinculin, which has been commonly used as a house-keeping gene. Mutation on Tyr at 6^th^ residue or Phe at 8^th^ residue affected biological activity of ADP355, suggesting that these two hydrophobic residues are important for AdipoR1 binding and activation. Among designed peptides during early stage, only BHD15 (*) showed higher cellular efficacy than ADP355.
(TIF)

S2 Fig SPR screening of designed peptides. ADP355, its analogues (Y6A and F8A), and the designed peptide candidates were flowed onto AdipoR1-Δ88 immobilized CM5 chip in Biacore T-200 to analyze their binding responses. Compared to ADP355, peptides were selected based on concentration-dependent signal increase, dissociation rate, and estimated Kd values. Among 25 candidate peptides, BHD15, BHD31 (re-named to BHD1028), BHD32, BHD33, BHD34, BHD43, BHD44, BHD45, and BHD46 were preliminarily selected.
(TIF)

S3 Fig Docking simulation between BHD1028 and AdipoR2. BHD1028 was simulated to bind to AdipoR1 (green backbone) and AdipoR2 (cyan backbone). For simulation for AdipoR2, 3WXW PDB ID was used. At the position of Gly364 in AdipoR1, AdipoR2 have Met375, which provides steric hindrance to the hinge region of BHD1028, suggesting that BDH1028 may have lower affinity to AdipoR2 than AdipoR1. Red stick indicates methionine side chain of AdipoR2 and yellow stick indicates side chains in the ligand-binding pockets of AdipoR1.
(TIF)
