# Supplementary figures and images for: Discovery of a novel potent peptide agonist to adiponectin receptor 1
Source: PLoS One. 2018 Jun 18;13(6):e0199256. doi: 10.1371/journal.pone.0199256 (PMC6005460; doi:10.1371/journal.pone.0199256)

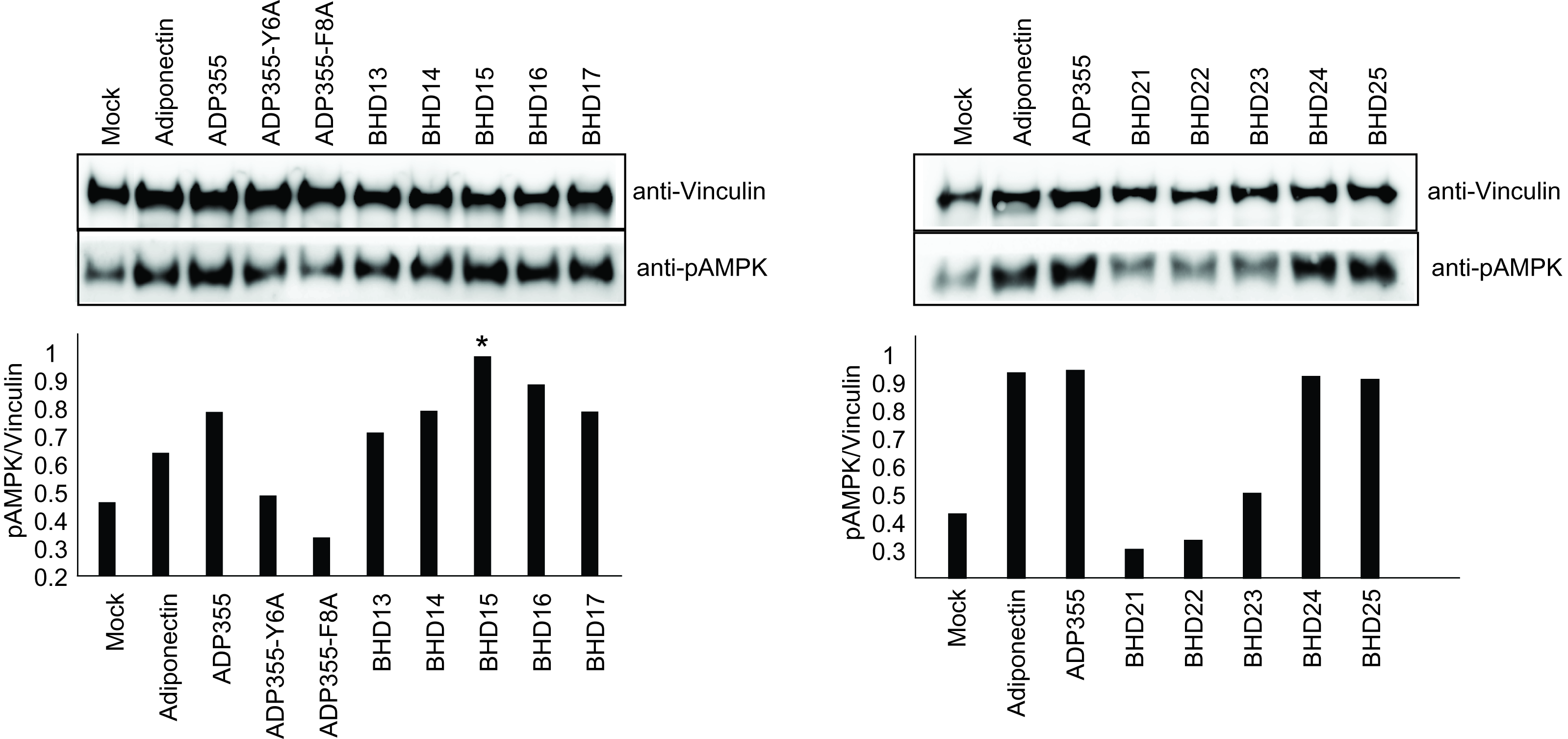

Supplement: S1 Fig — HepG2 cells were treated with 2 μg/ml globular adiponectin, 10 μM ADP355, ADP355-Y6A, ADP355-F8A and other designed peptide during early screening stage. Activation of AMPK was analyzed by Western blot with anti-pAMPK. Western signal of phospho-AMPK was normalized by that of vinculin, which has been commonly used as a house-keeping gene. Mutation on Tyr at 6th residue or Phe at 8th residue affected biological activity of ADP355, suggesting that these two hydrophobic residues are important for AdipoR1 binding and activation. Among designed peptides during early stage, only BHD15 (*) showed higher cellular efficacy than ADP355. (TIF) [file pone.0199256.s001.Tif]

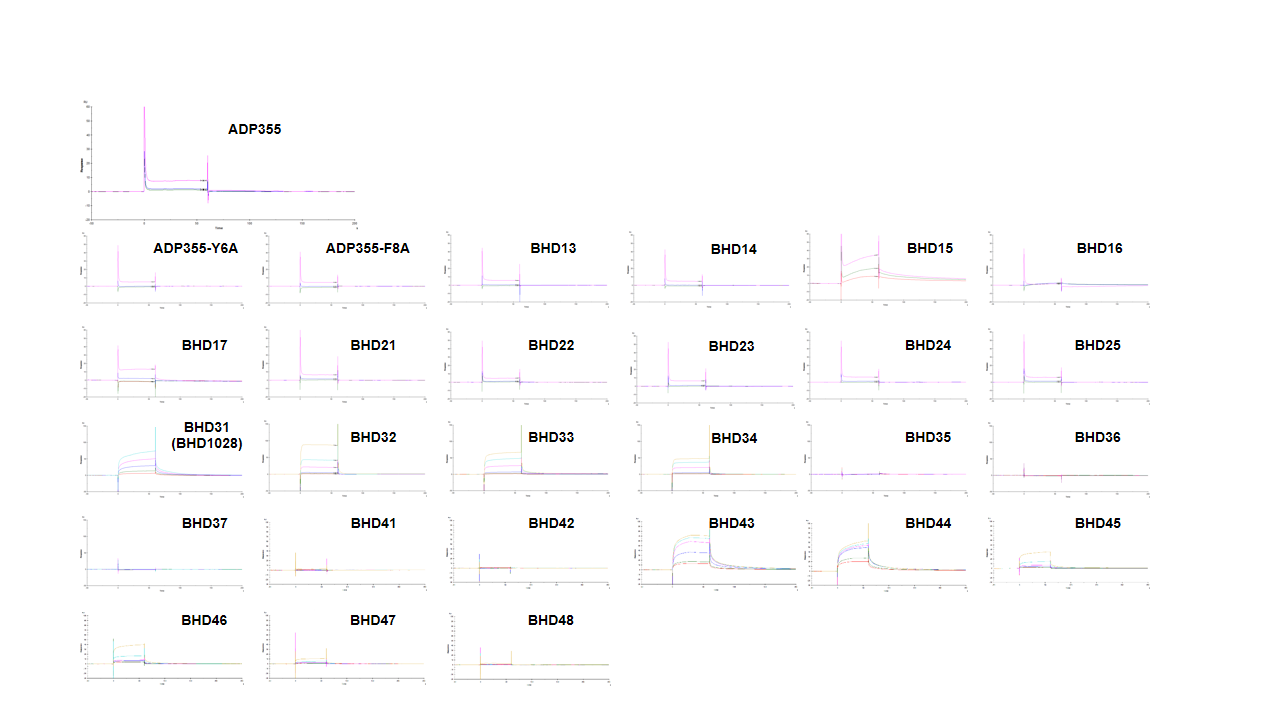

Supplement: S2 Fig — ADP355, its analogues (Y6A and F8A), and the designed peptide candidates were flowed onto AdipoR1-Δ88 immobilized CM5 chip in Biacore T-200 to analyze their binding responses. Compared to ADP355, peptides were selected based on concentration-dependent signal increase, dissociation rate, and estimated Kd values. Among 25 candidate peptides, BHD15, BHD31 (re-named to BHD1028), BHD32, BHD33, BHD34, BHD43, BHD44, BHD45, and BHD46 were preliminarily selected. (TIF) [file pone.0199256.s002.TIF]

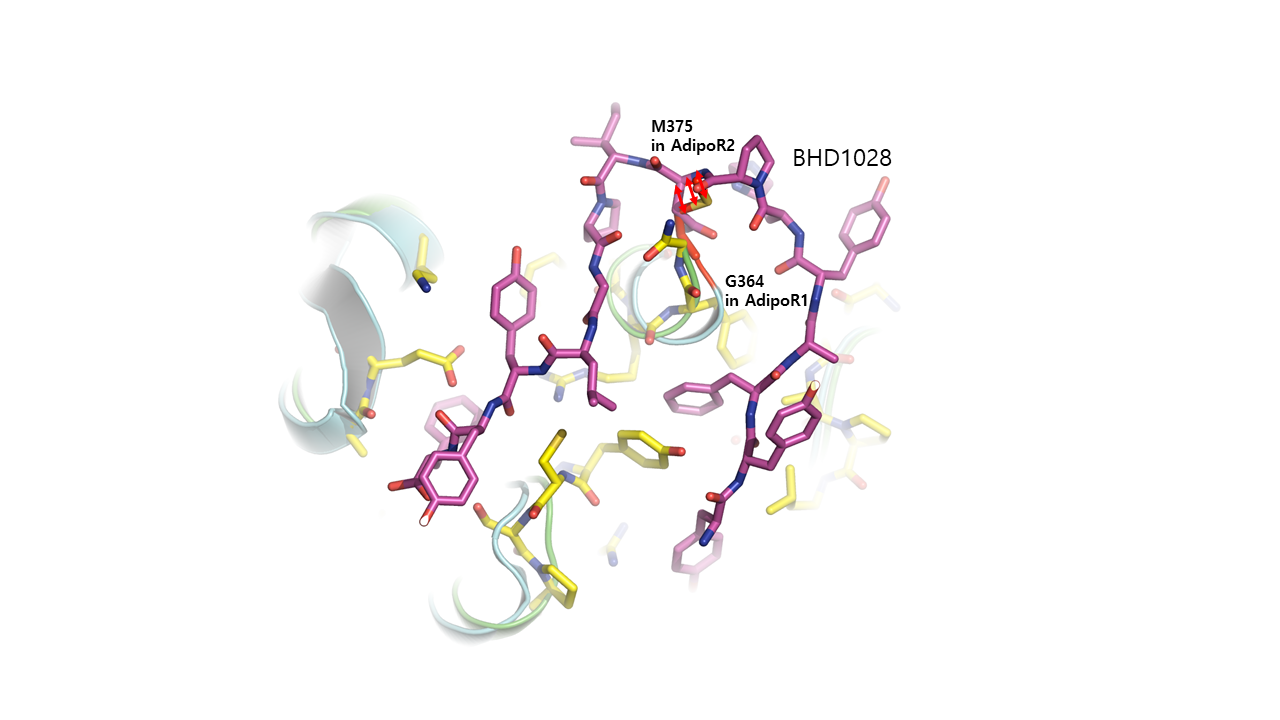

Supplement: S3 Fig — BHD1028 was simulated to bind to AdipoR1 (green backbone) and AdipoR2 (cyan backbone). For simulation for AdipoR2, 3WXW PDB ID was used. At the position of Gly364 in AdipoR1, AdipoR2 have Met375, which provides steric hindrance to the hinge region of BHD1028, suggesting that BDH1028 may have lower affinity to AdipoR2 than AdipoR1. Red stick indicates methionine side chain of AdipoR2 and yellow stick indicates side chains in the ligand-binding pockets of AdipoR1. (TIF) [file pone.0199256.s003.tif]
